# Considerations regarding the morphological variability of the superior cerebellar artery

**DOI:** 10.1007/s00234-025-03659-1

**Published:** 2025-06-03

**Authors:** Panagiotis Papadopoulos-Manolarakis, George Triantafyllou, Panagiotis Papanagiotou, George Tsakotos, Evangelia Christodoulou, Maria Piagkou

**Affiliations:** 1 Department of Anatomy, School of Medicine, Faculty of Health Sciences, National and Kapodistrian University of Athens, Athens, Greece; 2 Department of Neurosurgery, General Hospital of Nikaia-Piraeus, Athens, Greece; 3 Department of Radiology, Aretaieion University Hospital, School of Medicine, National and Kapodistrian University of Athens, Athens, Greece; 4 Department of Radiology, University Hospital of Patras, Patra, Greece

**Keywords:** Superior cerebellar artery, Variation, Origin, Morphology, Anatomy, Neuroradiology

## Abstract

**Purpose:**

The superior cerebellar artery (SCA) is a critical vascular structure supplying the cerebellum and brainstem. Despite its anatomical importance, variations in its origin and morphology remain underreported. This study aims to systematically assess the origin and morphological variations of the SCA using computed tomography angiography (CTA) in a large retrospective cohort.

**Methods:**

A total of 500 CTA brain scans (1,000 SCAs) were retrospectively analyzed. The study categorized the SCA based on its origin into five types and its morphology into five distinct patterns.

**Results:**

The most common SCA origin was from the distal basilar artery (BA) (65.3%), followed by a common trunk with the posterior cerebral artery (PCA) at the BA tip (24.5%) and an origin from the PCA (9.6%). A rare case of SCA arising from a fetal-type PCA (0.1%) was identified. Morphologically, the typical SCA pattern was observed in 92.9% of cases, while 7.1% exhibited variations. The most frequent morphological variant was complete duplication (4.7%), followed by early bifurcation (1.3%), fenestration (0.8%), and double origin (0.3%). No significant correlations were found between SCA variability and sex or laterality.

**Conclusions:**

This study provides a comprehensive classification system for SCA origin and morphology. Given the clinical relevance of these variations in neurosurgical and neurointerventional procedures, a thorough understanding of SCA anatomy is essential. The findings contribute to improved diagnostic accuracy and surgical planning, highlighting the need for precise imaging-based assessment of cerebrovascular anatomy.

## Introduction

The cerebral blood supply can be readily evaluated through various imaging modalities utilized in routine clinical practice, including computed tomography scan (CT), magnetic resonance imaging (MRI), and digital subtraction angiography (DSA). These imaging techniques provide critical anatomical information that may significantly influence clinical decisions made by healthcare professionals, including clinicians and surgeons [1, 2].

The cerebral arterial circle comprises several branches, with their anastomoses originating from the internal carotid and vertebrobasilar systems. The posterior circulation arises from the confluence of the two vertebral arteries (VAs), which form the basilar artery (BA), subsequently bifurcating into the posterior cerebral arteries (PCAs). The cerebellum receives its arterial supply from three primary arteries: the posterior inferior cerebellar artery (PICA) originating from the distal VA, the anterior inferior cerebellar artery (AICA) stemming from the proximal BA, and the superior cerebellar artery (SCA) arising from the distal BA. As stated in Gray’s Anatomy, the SCA courses beneath the oculomotor nerve, delineating the SCA and PCA, and also traverses below the trochlear nerve on its trajectory to its final course on the superior cerebellar surface [3].

Bergman’s Comprehensive Encyclopedia of Human Anatomic Variations notes a limited number of SCA variants, including vessel duplication (occurring with a prevalence of 14%) and the rare variant characterized by its absence [4]. Few studies document this vessel’s variant origins and morphologies [5]. Thus, the objective of the present imaging study was to explore the morphological variability of the SCA, including its origin and morphology, as well as their combinations and coexisting variants, such as the fetal type PCA, in a comprehensive retrospective study.

## Materials and methods

Five hundred (500) brain computed tomography angiographies (CTAs) were randomly selected and retrospectively analyzed for the SCA variants. The gender distribution included 312 males and 188 females, with a mean age of 59.5 ± 14.5 years. The CTAs were performed using a helical high-speed, low-dose scanner (SOMATOM go.Top, 128-slice configuration, Siemens) with the patient’s head in the supine neutral position, following the injection of 60 mL of a 30% iodine solution at a flow rate of 4–4.5 mL/s. The scans were obtained from the General Hospital of Nikaia-Piraeus, having received ethical approval from the appropriate authorities (protocol number: 56485, date of approval: 13.11.2024). Two authors independently reviewed the files (PPM, GTr), and any discrepancies were resolved by a third author, an expert neuroradiologist (PP). The study was conducted and documented utilizing the Horos software (Horos Project). Evidence was gathered regarding the multiplanar reconstruction of the axial, coronal, and sagittal slices, along with their respective three-dimensional volume reconstruction.

**Fig. 1:**
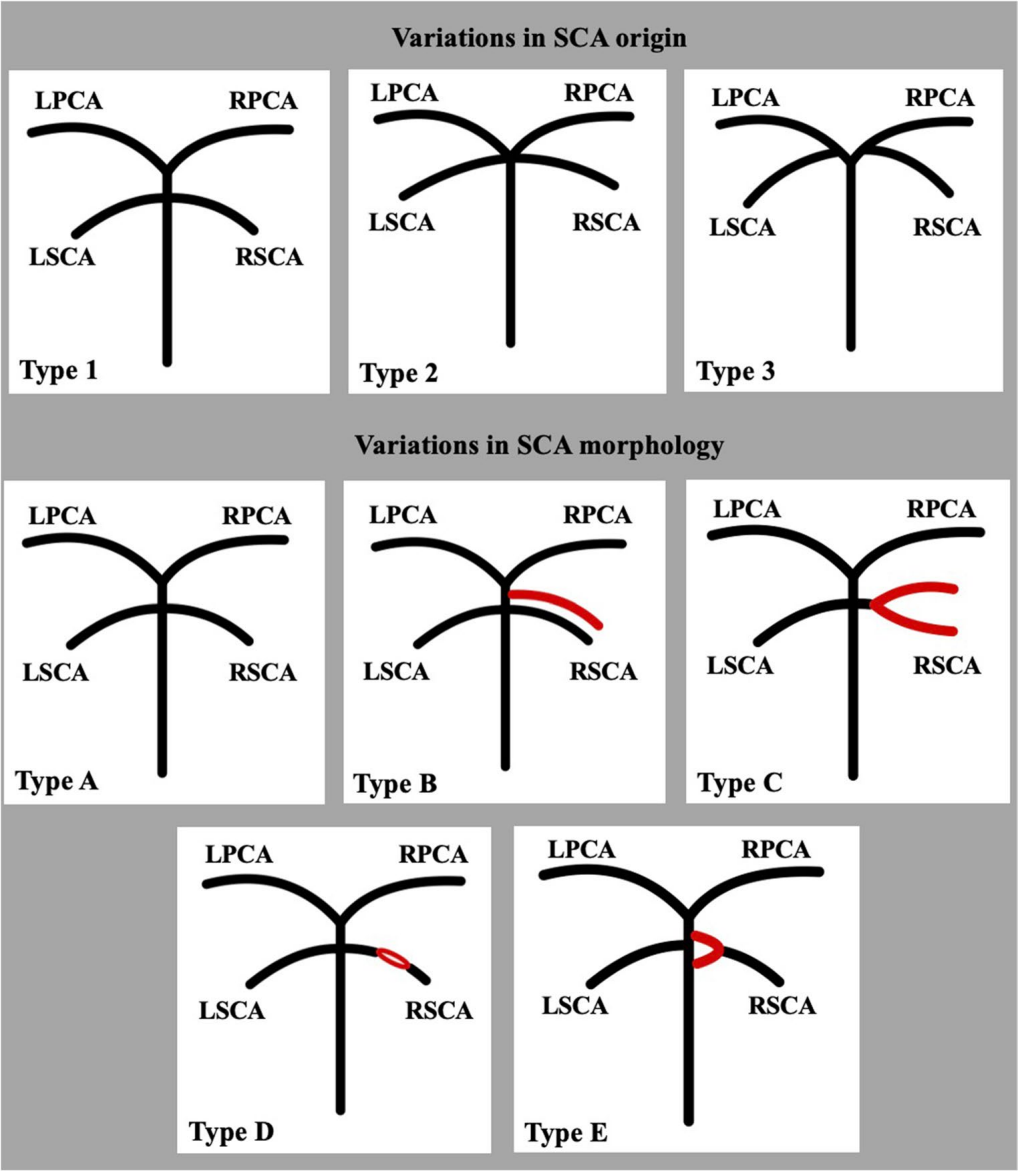
Schematic representation of the superior cerebellar artery (SCA) variations according to its origin and morphology. Variants of SCA origin: Type 1- origin from the distal basilar artery (BA); Type 2- origin from the BA tip in common with the posterior cerebral artery (PCA); Type 3- origin from the PCA. Variants of the SCA morphology: Type A- typical morphology; Type B- SCA duplication; Type C- SCA early bifurcation; Type D- SCA fenestration; Type E- SCA duplicate origin

The SCA variants were assessed based on their origin and morphology, as summarized in schematic representation (Fig. 1). Three distinct origin patterns of the SCA were recorded in cases in which the PCA typically originated from the BA: SCA type 1 corresponded to the origin from the distal BA (collateral pattern); SCA type 2 corresponded to the SCA emanated from the BA terminal part (the so-called tip of the BA), in common with the PCA (terminal pattern); and SCA type 3 represented the origin of the SCA from the PCA (cerebral pattern).

Nevertheless, the vessel’s absence was categorized as SCA type 0. Moreover, in cases where the PCA exhibited a fetal origin (FPCA) with an absent P1 segment (complete FPCA), the SCA could originate from the BA tip as a terminal branch, classified as SCA type 2, or it could emanate from the FPCA of the internal carotid artery (ICA) system, classified as type 4.

Five morphological patterns of the SCA were identified: typical (type A), complete duplication (two distinct SCAs on one or both sides) (type B), early bifurcation (type C), fenestration (refers to a segment of the artery where it splits into two parallel channels that rejoin distally) (type D), and double origin of the vessel (type E) (Fig. 1).

Statistical analysis was conducted using IBM SPSS Statistics for MacOS, Version 29 (IBM Corp., Armonk, New York, United States). The Chi-square test was used to compare nominal data between unpaired observations, while McNemar’s was used for paired observations. A p-value of equal or less than 0.05 was deemed statistically significant.

## Results

**Fig. 2:**
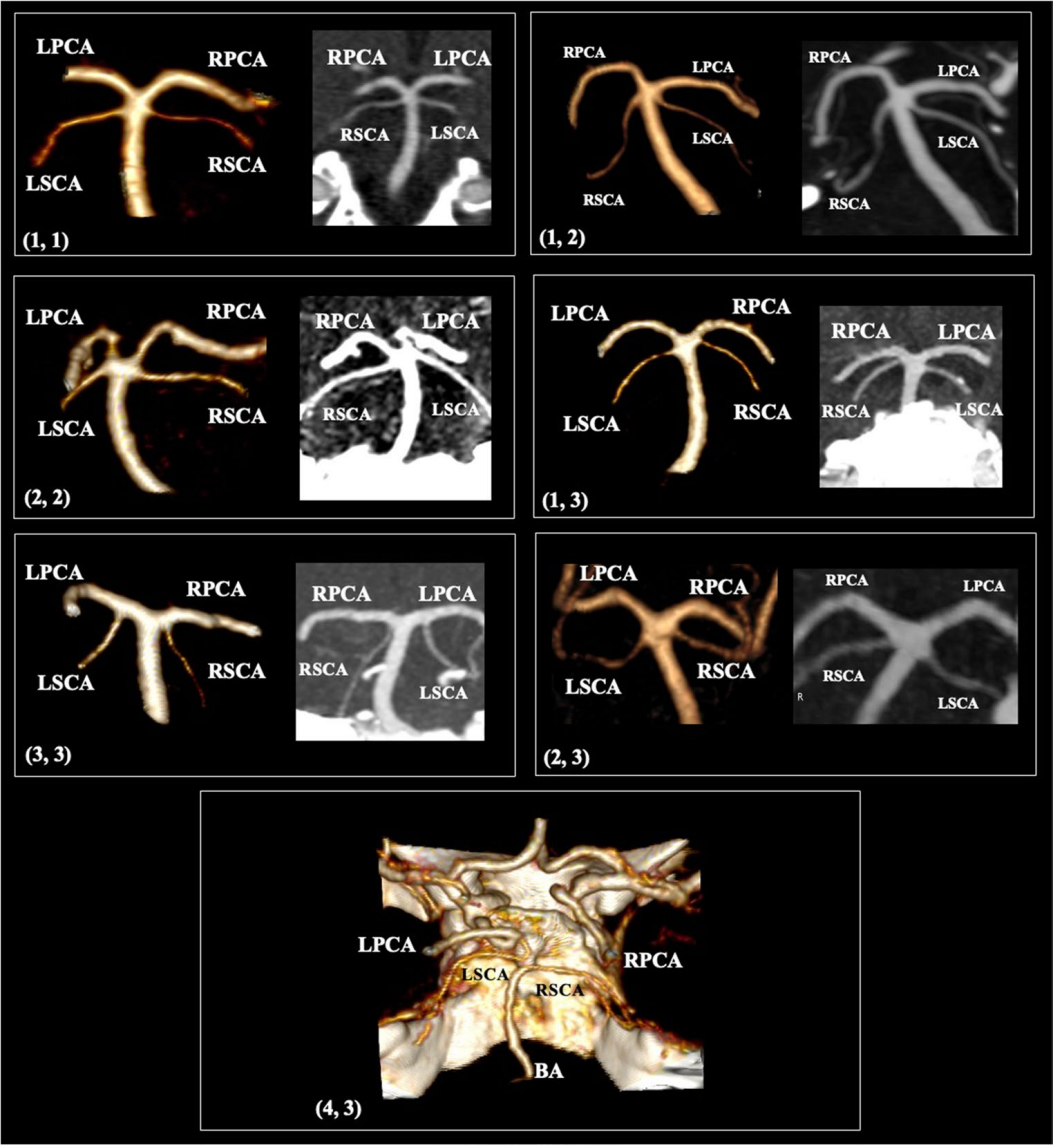
Anatomical possibilities of the superior cerebellar artery (SCA) origin. Three-dimensional reconstruction and coronal sections. The SCA origin types are reported on the left corner of each figure

The most common SCA origin was from the distal BA (*type 1- collateral pattern*) identified in 65.3% of vessels, followed by the common origin with the PCA from the BA tip (ending point) (*type 2- terminal pattern*) observed in 24.5%. In 9.6% of cases, the SCA emanated from the PCA (*type 3- cerebral pattern*). The absence of the vessel was recorded at 0.5%. A unique case corresponded to an SCA origin from an FPCA (ICA system) with 0.1% prevalence (Fig. 2). The side and sex did not influence the SCA origin (Table 1). Symmetrical SCA origin (bilateral the same type) was observed at 57.8%. Bilateral type 1 was identified in 46.6% (233 patients), type 2 in 9.4% (47 patients), and type 3 in 1.8% (9 patients). Therefore, 42.2% of patients had an asymmetrical SCA origin. The most common combination was type 1 and 2 observed in 127 patients (25.4%), and the rarest combination was type 3 and 4 in one patient (0.2%).

**Table 1:** Morphological variability of the superior cerebellar artery (SCA) origin pattern

SCA Origin	Total (*n* = 1000)	Left (*n* = 500)	Right (*n* = 500)	*p*-value	Males (*n* = 624)	Females (*n* = 376)	*p*-value
Type 0	5 (0.5%)	2 (0.4%)	3 (0.6%)	0.862	3 (0.4%)	2 (0.5%)	0.284
Type 1	653 (65.3%)	332 (66.4%)	321 (64.2%)		417 (66.8%)	236 (63%)	
Type 2	245 (24.5%)	116 (23.2%)	129 (25.8%)		146 (23.4%)	99 (26.3%)	
Type 3	96 (9.6%)	49 (9.8%)	47 (9.4%)		58 (9.3%)	38 (10.2%)	
Type 4	1 (0.1%)	1 (0.2%)	0 (0%)		1 (0.1%)	0 (0%)	

**Fig. 3:**
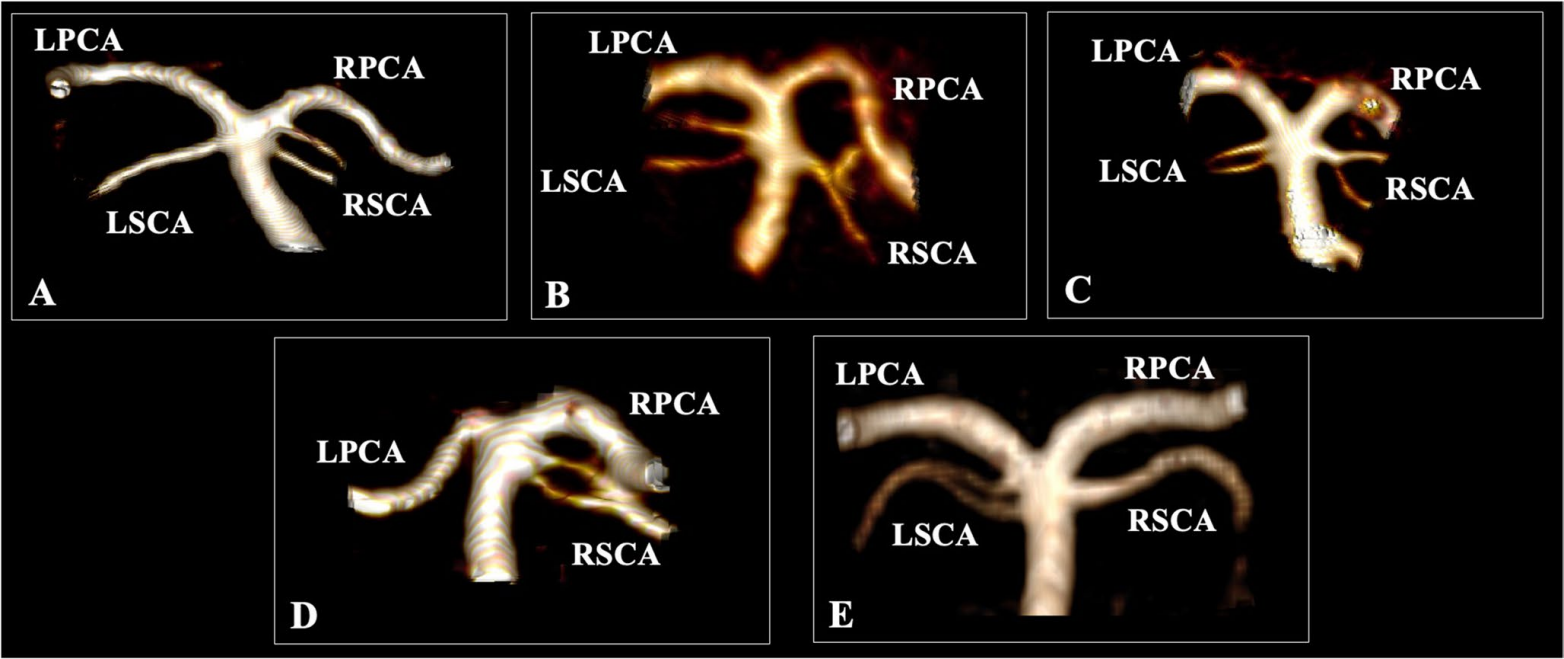
Anatomical possibilities of the superior cerebellar artery (SCA) morphology. Three-dimensional reconstruction. (**A**) Duplication (SCA type 1 and 3); (**B**) Duplication (both SCAs type 1); (**C**) Left-sided duplication and right-sided early bifurcation; (**D**) Fenestration; (**E**) Duplicate origin

A typical SCA morphology was identified in 92.9%; thus, variations were observed in 7.1% of cases. The most common morphological variant was the SCA complete duplication identified in 4.7%. The accessory vessel could originate from the distal BA (3.2%), the BA tip (1%), or the PCA (0.5%). An early bifurcation was recorded in 1.3%, while SCA fenestration was observed in 0.8%. Lastly, the rarest variation was the double origin of the vessel at 0.3% (Fig. 3). The side and sex did not influence the SCA morphological variability (Table 2). Bilateral duplication was recorded in 0.6% (3 patients) and bilateral fenestration in 0.2% (1 patient). All the rest of the morphological variations were recorded unilaterally.

**Table 2:** Morphological variability of the superior cerebellar artery (SCA) morphology pattern

SCA Morphology	Total (*n* = 1000)	Left (*n* = 500)	Right (*n* = 500)	*p*-value	Males (*n* = 624)	Females (*n* = 376)	*p*-value
Typical	929 (92.9%)	468 (93.6%)	461 (92.2%)	0.156	575 (92.1%)	344 (91.5%)	0.561
Duplication	47 (4.7%)	23 (4.6%)	24 (4.8%)		23 (3.7%)	24 (6.3%)	
Early Bifurcation	13 (1.3%)	4 (0.8%)	9 (1.8%)		9 (0.1%)	4 (1.1%)	
Fenestration	8 (0.8%)	4 (0.8%)	4 (0.8%)		4 (0.6%)	4 (1.1%)	
Double Origin	3 (0.3%)	3 (0.6%)	0 (0%)		3 (0.5%)	0 (0%)	

When correlating the SCA origin patterns with the morphological variations, we identified that morphological variations occurred statistically more commonly when the SCA originated from the distal BA (type 1). Specifically, out of the 71 cases with morphological variations, 64 had SCA type 1, 6 had SCA type 2, and 1 had type 3. Table 3 summarizes a detailed table of the combinations between SCA origin patterns and morphological variations.

**Table 3:** Correlation of the type of origin and morphological variant of the superior cerebellar artery (SCA) on the current sample. The results are presented based on the number of cases

SCA Origin	Typical (*n* = 929)	Duplication (*n* = 47)	Early Bifurcation (*n* = 13)	Fenestration (*n* = 8)	Double Origin (*n* = 3)
Type 0 (*n* = 5)	5	0	0	0	0
Type 1 (*n* = 653)	589	47	7	7	3
Type 2 (*n* = 245)	241	3	0	1	0
Type 3 (*n* = 96)	92	0	4	0	0
Type 4 (*n* = 1)	1	0	0	0	0

One hundred and three patients (20.6%) had a fetal PCA: 16 patients had bilateral occurrence (3.2%), 41 patients had it unilaterally on the left side (8.2%), and 46 patients unilaterally on the right side (9.2%). These patients had a significantly higher frequency of type 2 compared to the patients without fetal type PCA (*p* < 0.001).

## Discussion

The current extensive retrospective imaging-anatomical study (500 patients with *n* = 1000 arteries) assessed the SCA morphological anatomy through CTAs in a Greek adult population. After analyzing this large retrospective sample, we observed five patterns for the SCA origin and five SCA morphological patterns. Using the proposed classification, the SCA variants and their combinations could be easily described (Fig. 1). Although the embryology of the vascular anatomy of the brain has been extensively studied, there are only limited data about the SCA development. Variations of the vessels seem to arise from an abnormal fusion of the caudal end of the BA. For example, a fusion between the PCA and the BA at a lower point than typical could explain the PCA origin of the SCA [6].

Even though anatomical textbooks do not refer to the high variability of the SCA origin [3, 4], there are few anatomical possibilities. Three possible patterns are commonly referred to as the SCA origin, as depicted in Fig. 1. In their review, Malicki et al. [5] reported the typical SCA origin (type 1 according to the present study) from the distal BA, ranging between 70.7 and 89.43%. In comparison, a cadaveric study with only 25 specimens reported a prevalence of 35% [7]. Davidoiu et al. [8] reported type 1 origin in 71.29%. In the present study, we reported a prevalence of 65.3%, close to the previous imaging studies. The slight difference could be attributed to the different populations or the larger sample of our research. Malicki et al. [5] reported the common origin with the PCA ranging between 1 and 5%, while a cadaveric study reported it in 40% of their sample [7]. Davidoiu et al. [8] recorded a higher prevalence of 19.06%, closer to the current results, identified a prevalence of 24.5%. Lastly, Malicki et al. [5] identified a prevalence between 2 and 15% for the SCA origin from the PCA. This type 3 origin was recorded in 9.41% of cases in Davidoiu et al. [8] % in the study and 9.6% in our study. Nevertheless, we identified a single peculiar case of SCA originating from a FPCA (emanating from the ICA). Malicki et al. [5] did not report a similar case during their literature review. However, we identified a previously published case. Alnafie [6] described a case of bilateral SCA duplication, while the right-sided accessory SCA emanated from a fetal PCA. Uchino et al. [9] and Shoja et al. [10] independently reported another unique case of SCA originating from the ICA (indicative of persistent trigeminal artery-PTA). Therefore, except for the three anatomical possibilities that are frequently reported [5, 8], we are suggesting an additional type 4 for the SCA origin. Furthermore, Elzawawy et al. [11] identified a case of hypoplastic right vertebral artery associated with the partial duplication of the distal segment of the right P1 segment of a FPCA and bilateral duplication of the SCA, wherein the upper right SCA originated from the PCA. The authors postulate that the inadequate development of the right half of the vertebrobasilar system contributed to the persistence of the FPCA with an anomalous origin of the right upper SCA, along with the partial duplication of the P1 segment of the PCA, which may serve as a remnant of the deficient anastomosis between the embryonic right PCA and the basilar system. Such intricate variations present significant challenges in diagnosis and the selection of appropriate treatment modalities for the condition of stroke [11].

SCA variants are essential for neuroradiologists and neurosurgeons in various conditions and operations, such as vascular malformations, distal/terminal BA lesions, trigeminal neuralgia, and BA termination variants during ligation, temporal lobectomies, and posterior cerebral revascularization [8]. Our study shows that various SCA origin possibilities can be easily diagnosed during diagnostic and interventional neuroradiology procedures with CTA, MRA, and DSA. Cerebellar infractions caused by SCA thrombotic or embolic occlusions can cause a broad spectrum of clinical symptoms, such as rostral BA syndrome and vestibulocerebellar syndrome [8]. Nevertheless, a thorough understanding of the BA tip anatomy is paramount during microneurosurgical and endovascular/neurointerventional approaches. Vascular details and topographical relationships are helpful for both neurosurgeons and interventional neuroradiologists.

The morphological variability of the SCA is frequently identified as a duplicated vessel. We identified duplicated SCAs in 4.7% of cases. Nevertheless, in the current study, we recorded the accessory SCA origin type, type 1 being the most common. In the literature review, Malicki et al. [5] recorded duplicated SCAs in 3.61–31% of cases [5]. However, the origin patterns of the accessory SCA were not reported previously. The SCA early bifurcation was identified in 3–9.4% of the cases, according to previous studies [5], while our study revealed a prevalence of 1.3%. SCA fenestration could be considered a rare entity. Davidoiu et al. [8] identified this variant in 0.48% of cases, with the present study reporting a prevalence of 0.8%. Sonoda et al. [12] described a case of SCA identified during MRA and DSA, while they mentioned that their case was only the seventh reported in the current literature. Moreover, we described a novel variation of the SCA as a double origin previously reported for the anterior and middle cerebral arteries [13]. This variant consists of two vessels originating from the BA that merge to form a single vessel shortly after their origin. We identified a prevalence of 0.3% for this variation. Porzionatto et al. [14] described the occurrence of a hemorrhagic infarction of the cerebellum in the vascular field of an SCA with double origin as intriguing, suggesting a possible pathophysiological association.

SCA variants may sometimes be combined with other regional arterial variants. Endo et al. [15] reported the coexistence of an early bifurcation of the SCA alongside a persistent trigeminal artery, a duplicated AICA, and a vertebral artery originating from the aortic arch. Uchino et al. [16] identified a case in which a right-replaced PCA was associated with an ipsilateral SCA-type persistent trigeminal artery variant.

**Fig. 4:**
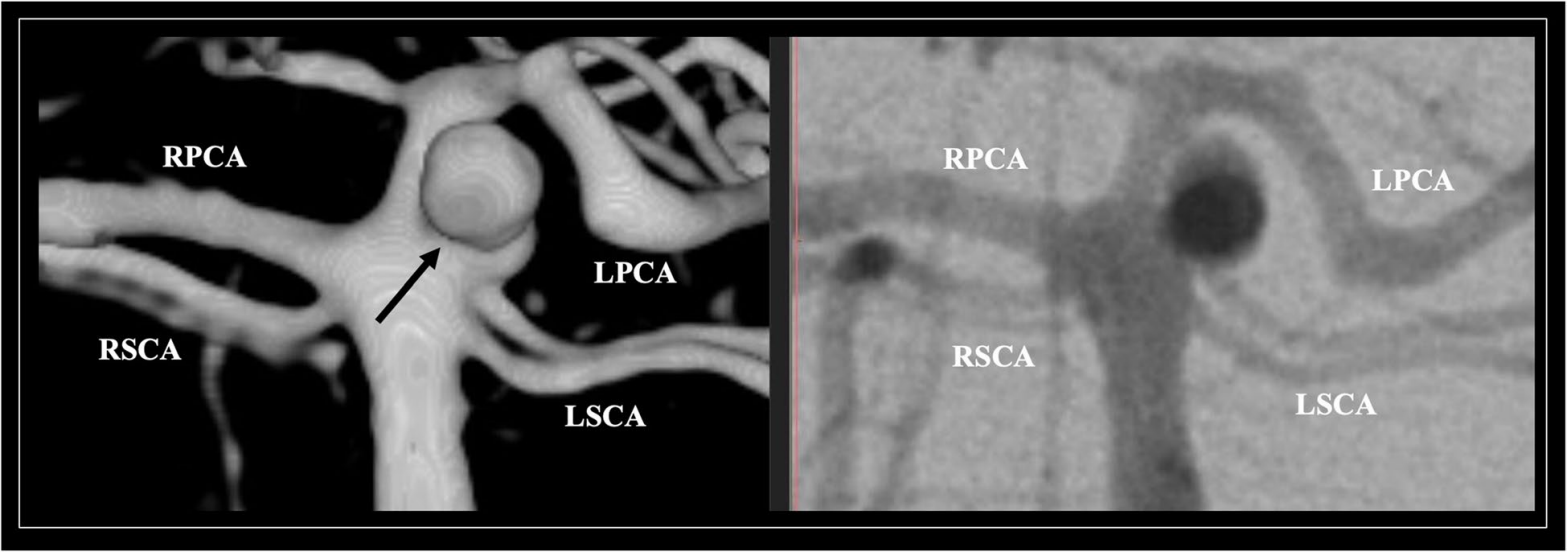
An illustrative case of an aneurysm (arrows) located at the origin of the superior cerebellar artery (SCA) depicted on digital subtraction angiography (DSA) and its three-dimensional reconstruction. PCA- posterior cerebral artery

The morphological variants could contribute to the formation of an SCA aneurysm due to the altered hemodynamics caused by these variants, similar to other cerebral vessels. Although SCA aneurysms are rare, they typically form at the junction with the BA (Fig. 4). Surgical treatment is associated with favorable outcomes; however, the anatomical relationship with critical structures (major perforating arteries and adjacent cranial nerves) could complicate neurosurgical procedures [8]. The SCA can be implicated during BA aneurysm repair, which is a frequent challenge for microneurosurgical treatment. Knowledge of the anatomical details, such as the SCA origin from the BA tip, is essential preoperatively either for direct clipping or coil embolization [8].

It is important to mention a few significant details about the SCA’s topographical variability. Rusu et al. [17] described the topographical relationships between the SCA pontomesencephalic segment and the trochlear nerve during cadaveric dissection. Several possibilities were identified: the trochlear nerve coursed above the primary trunks of the SCA and then below the main trunk of the artery, compressed between the SCA and the brainstem, and through a ring formed by the SCA [17]. Additionally, Tsutsumi et al. [18] described the relationship of the oculomotor nerve between the SCA and PCA during MRA scans. They identified the nerve in contact with the SCA in 7 cases (9.9%) but did not observe any compression cases (0 cases, 0%). As mentioned before, the topography with the cranial nerves is significant when treating SCA aneurysms with neurosurgical operations. Kuniak et al. reported a paresis of the oculomotor nerve due to neurovascular conflict with the SCA [19]. Based on the SCA segments and their relationship with cranial nerves, different clinical symptoms can be caused such as trigeminal neuralgia, oculomotor compression syndromes [5]. Trigeminal neuralgia can be frequently caused by compression of the nerve by the caudally coursed SCA. Patients with duplicated or early bifurcated SCA have a higher risk of trigeminal neuralgia [9].

The current study has a few limitations. Although the sample size was the largest among studies investigating SCA variability (*n* = 1000 cases), the rarer morphological variations were identified in only a few instances, making it challenging to perform statistical analysis to depict possible correlations. Nevertheless, this sample was derived from a specific population in Athens, Greece. We encourage researchers to investigate the origin and morphology of SCA further by using the current classification system. Additionally, employing other techniques, such as MRA or cadaveric dissection, will enhance our understanding of topographical variability.

## Conclusions

The current imaging-anatomical study described the SCA origin patterns and morphological variations. We identified that the most common origin of the vessel was the distal BA in 65.3% and its typical morphology presented in 92.9%. The most common variant origin was in a common trunk with the PCA in 24.5%, while its common morphological variant was the complete duplication in 4.7%. Based on the large retrospective analysis that it was performed (*n* = 1000 cases), the SCA has a significant variability that it should be carefully considered when planning or performing operations in the region. Lastly, we are proposing a simple classification system for the categorization of all these variants.

## Data Availability

No datasets were generated or analysed during the current study.
